# Cytokine Signature and Involvement in Chronic Rhinosinusitis with Nasal Polyps

**DOI:** 10.3390/ijms23010417

**Published:** 2021-12-30

**Authors:** Florent Carsuzaa, Émilie Béquignon, Xavier Dufour, Guillaume de Bonnecaze, Jean-Claude Lecron, Laure Favot

**Affiliations:** 1 Laboratoire Inflammation Tissus Epithéliaux et Cytokines (LITEC), Université de Poitiers, 86000 Poitiers, France; xavier.dufour@chu-poitiers.fr (X.D.); jean-claude.lecron@univ-poitiers.fr (J.-C.L.); laure.favot@univ-poitiers.fr (L.F.); 2 Oto-Rhino-Laryngologie et Chirurgie Cervico-Maxillo-Faciale, Centre Hospitalier Universitaire de Poitiers, 86021 Poitiers, France; 3 Oto-Rhino-Laryngologie et Chirurgie Cervico-Faciale, Hôpital Henri Mondor et Centre Hospitalier Intercommunal de Créteil, 94010 Créteil, France; emilie.bequignon@gmail.com; 4 INSERM U955, Équipe 13, Centre Henri Mondor de Recherche Biomédicale, 94000 Créteil, France; 5 Oto-Rhino-Laryngologie et Chirurgie Cervico-Faciale, Centre Hospitalier Universitaire de Toulouse, 31400 Toulouse, France; debonnecaze.g@chu-toulouse.fr; 6 Service Immunologie et Inflammation, Centre Hospitalier Universitaire de Poitiers, 86021 Poitiers, France

**Keywords:** chronic rhinosinusitis, cytokines, inflammation, nasal polyps, interleukin, epithelial cell

## Abstract

Cytokines are well known to play a central role in chronic rhinosinusitis with nasal polyps (CRSwNP), particularly in maintenance of the inflammatory response and the recruitment of eosinophils. The pathophysiological concepts concerning the involvement of inflammatory cytokines in CRSwNP have gradually evolved. Although the Th2 cytokines environment associated with an eosinophilic infiltration has retained a central role in the genesis of polyps, the role of other cytokine subpopulations has also and more recently been detailed, leading to a specific and complex signature in CRSwNP. The purpose of this review is to summarize the current state of knowledge about the cytokine signature in CRSwNP, the role of cytokines in the pathogenesis of this disease and in the intercellular dialog between epithelial cells, fibroblasts and inflammatory cells. Knowledge of this precise cytokine signature in CRSwNP is fundamental in the perspective of potential targeting biotherapies.

## 1. Introduction

Chronic rhinosinusitis with nasal polyps (CRSwNP) or without nasal polyps (CRSsNP) are inflammatory diseases of the nasal mucosa and paranasal sinuses. In this review, we focus on CRSwNP that affects 4% of the general population. Symptoms are related to the bilateral and multifocal development of polyps and include nasal obstruction, hyposmia, discharge and facial pain or pressure, which lasts for at least 3 months and causes substantially impaired quality of life [1]. Medical treatment is based on topical or systemic corticosteroids. Surgery is performed in patients having failed well-conducted medical treatment. Although effective, surgery does not prevent recurrence of symptoms related to polyp regrowth. Recent biotherapies, including anti-IgE, anti-IL-5 and anti-IL-4Rα, are of demonstrated effectiveness, and their use has grown exponentially [2].

Typical histological features of nasal polyps include inflammation of type 2 T-helper cells (Th2) accompanied by major tissue remodeling (thickening of basement membrane, hyperplasia of the epithelium, fibrosis). The dense inflammatory infiltrate is composed of several immune cell types such as T and B lymphocytes, group 2 innate lymphoid cells (ILC2), eosinophils and neutrophils, mast cells and macrophages. The cytokines produced by these immune cells and a number of other cells (epithelial cells or fibroblasts) play a central role in the pathogenesis of inflammatory diseases, including CRSwNP. Cytokines are key factors generating and modulating cell-to-cell communication in immunological responses, hematopoietic development as well as host responses to infectious agents and inflammatory stimuli. 

CRSwNP has long been considered to be an eosinophil-dominant Th2 inflammatory disease, and eosinophil inflammation represents a hallmark of Th2 skewing. However, the relevant concepts have gradually become more sophisticated, and the situation has become, more complex with description of the involvement of other T-lymphocyte subpopulations (Th17, Th22) in CRSwNP. 

The concept of endotype progressively emerged and was defined according to the cytokines signature found in different group of patients: (a) markers of eosinophilic and Th2 driven inflammation, (b) neutrophilic and proinflammatory cytokines, (c) Th17- or Th22-related markers and (d) Th1 inflammatory markers. Endotypes are present with a wide diversity of inflammatory profiles and strongly correlated with characteristic phenotypes observed in patients with CRS. The expression of Th2 cytokines is the paramount profile dictating the phenotype with eosinophilic nasal polyps. However, one cluster of CRSwNP endotypes is characterized by a non-eosinophilic infiltration of polyps associated to a Th17 neutrophil-type inflammation, corresponding in some Asian patients. CRSwNP inflammation should now be considered multidimensionally heterogeneous on the Th1, Th2, Th17, eosinophilic/neutrophilic, proinflammatory, superantigen, and possibly TH22 axes [3]. 

Little is known about the events at the origin of CRSwNP. Infectious agents are thought to cause damage to the nasal epithelium, and the role of *Staphylococcus aureus* is to date the most documented. It produces toxins acting similar to superantigens and amplifying the inflammatory Th2 lymphocyte response, which is responsible for the recruitment and activation of eosinophils [4]. The role of fungal agents such as *Alternaria* has also been reported. *Alternaria* facilitate the production of antimicrobial molecules (lysozymes, complement fractions, defensins) and the release of pro-inflammatory cytokines (interleukin (IL)-1α, IL-33, thymic stromal lymphopoietin (TSLP) and tumor necrosis factor-α (TNF-α)), as well as adhesion factors for neutrophils and eosinophils [5,6].

Cytokines therefore play a central and essential role in the genesis of CRSwNP, particularly in the maintenance of the inflammatory response and the recruitment of eosinophils. Many of them also directly target fibroblasts and nasal epithelial cells by modifying their functions (epithelial barrier, differentiation, cell repair). 

The purpose of this review is to summarize the current state of knowledge about the cytokine signature in CRSwNP, the role of cytokines in the pathogenesis of this disease and in the intercellular dialog between epithelial cells, fibroblasts and inflammatory cells. The cytokines related to CRSwNP will be clustered as classical groups or families (Table 1). 

## 2. Type 2 Inflammatory Cytokines and TSLP

Th2 cells mediate the activation and maintenance of the humoral immune response against extracellular parasites, bacteria, allergens and toxins, driven particularly by IL-4, IL-5, IL-9 and IL-13. They are responsible for strong antibody production, eosinophil activation and counteract the Th1 response. Th2 pathway cytokines have a central place in the pathophysiology of CRSwNP, and IL-4 and IL-13 are the most widely described in this context. They are secreted not only by Th2 lymphocytes, but also by ILC2, eosinophils, basophils, natural killers and mast cells, exerting their action by binding receptors sharing the IL-4Rα chain. Type 2 inflammatory cytokines are found in nasal polyps and also in the associated non-polypoid sinonasal mucosa, demonstrating an extended type 2 inflammatory phenomenon [61]. The importance of these cytokines in the pathogenesis of CRSwNP is strengthened by the dramatic clinical responses to dupilumab, a fully humanized monoclonal antibody blocking IL-4Rα.

### 2.1. Interleukin 4 

IL-4 messenger ribonucleic acid (mRNA) and protein are overexpressed in nasal polyps. They stimulate the development of naïve Th0 lymphocytes into differentiated Th2 lymphocytes and prevent apoptosis of activated T cells. IL-4 enables the activation of B-cells and the production of immunoglobulins (Ig). Ig may play an important role in the inflammatory reaction of CRSwNP. IgA could make it possible to limit microbiological colonization [62] and IgD might intervene in the defense against respiratory bacteria [63]. Regarding the cytokine profile, IL-10 and TGF-β are involved in IgA switch and synthesis, and IL-4 in IgD synthesis [7]. In addition, IL-4 is involved in isotype switching from immunoglobulin IgM/IgG to IgE, which is believed to be a major feature of inflammation during CRSwNP [8,9]. IL-4 also promotes eosinophil migration through increased expression of vascular cell adhesion molecule (VCAM)-1 on human endothelial cells, and through the release the chemokine MCP-4 (CCL13), an important chemoattractant of eosinophils as documented by its production in supernatant of cultured nasal polyp fibroblasts stimulated with Toll-like receptor (TLR) 2, 3, 4 and 5 ligands [10]. This suggests a mechanism through which TLR ligands and IL-4 contribute to eosinophilic infiltrates of nasal polyps. IL-4, in combination with lipopolysaccharide, has also been shown to induce fibroblast release of chemokine (C-C motif) ligand 17 (CCL17), a potent chemoattractant for Th2 cells [64]. These results suggest that fibroblasts may be involved in the maintenance of chronic Th2-biased inflammation in nasal polyps. IL4 also plays a role in tissue remodeling through a profibrotic process, inducing changes in mRNA and protein expression of fibrotic factors by fibroblasts [11]. Regarding epithelial cells, IL-4 increases permeability by altering cells tight junctions [12] and decreases wound closure [13].

### 2.2. Interleukin 13

IL-13 is a pleiotropic cytokine associated with the development of polyps, and able to initiate the recruitment of inflammatory cells, the differentiation of monocytes into macrophages, and the activation of eosinophils [14,15,17]. IL-13 shares the same cellular receptor as IL-4, namely IL-4Rα. Its effects are therefore close to those of IL-4. IL-13 causes goblet cell hyperplasia in epithelial cells and proliferation of smooth muscle cells [16,65]. IL-13-mediated stimulation of the hypoxia signaling pathway has an anti-inflammatory effect mediated via CD73, a membrane-bound glycoprotein that assists in the metabolism of the “danger signals” ATP, ADP and AMP into anti-inflammatory adenosine [66]. Sinonasal epithelial cells exposure to IL-13 results in alteration of in intercellular junction proteins, reflecting increased epithelial permeability [12] but has no effect on epithelial repair [13].

### 2.3. Interleukin 5

Several studies have reported increased IL-5 mRNA and protein in nasal polyp tissue [8,18,19,67,68]. IL-5 plays a central role in chronic inflammatory response in nasal polyposis by inducing the accumulation of a large quantity of eosinophils within the polyps [68,69]. IL-5 is produced by Th2 cells, masts cells, innate lymphoid cells and eosinophils. The main source of IL-5 is believed to be eosinophils themselves, activating an autocrine loop necessary for their activation and survival [20]. IL-5 serum levels are elevated in all the endotypes of CRSwNP, but conversely not in CRSsNP endotypes. IL-5 is then suggested to be the key factor dictating the CRSwNP phenotype [3]. 

This cytokine has a pro-inflammatory action by attracting, activating and inducing the survival of eosinophils [18,19,21,22]. IL-5 also induces the activation, proliferation and differentiation of B-cells. B-cells, thereby activated, synthesize IgE and induce the proliferation of eosinophils [23]. Eosinophils contribute to the development and maintenance of the inflammatory reaction. They release mediators (major basic protein, eosinophilic cationic protein, eosinophil-derived neurotoxin, eosinoperoxidase) leading to lesions of the epithelium causing tissue remodeling [24]. They also produce signals that promote the mobilization of other actors in immune response such as CCL23 leading to the recruitment of macrophages, and IL-5 promoting the production of IgE by plasma cells. On epithelial cells, IL-5 has no effect on wound closure [13] and up until now its role on epithelial permeability in CRSwNP has not been studied.

### 2.4. Interleukin 9

IL-9 is a cytokine produced by mast cells, natural killer cells and two T-helper cell types, i.e., Th9 and Th2 cells [70]. IL-9 is involved in anti-parasitic and anti-tumor responses, and also in the formation of immune tolerance.

IL-9 mRNA and protein are overexpressed in nasal polyps compared to non-inflammatory nasal mucosa. In patients with associated asthma, IL-9 together with IL-5 induces IL-5Rα expression in airway eosinophils and promotes the anti-apoptotic action of IL-5 [25,71]. In addition, IL-9 is involved in the release of IgE by B-cells and in the production of proteases by mast cells leading to epithelial cell damage [26]. IL-9 also plays a direct role on the epithelial cell favoring muciparous metaplasia on bronchial cells [27].

### 2.5. Thymic Stromal Lymphopoietin

TSLP, an IL-7-like cytokine, is expressed by epithelial cells and plays a role in inflammation of allergic origin by stimulating dendritic cells, monocytes and T-helper lymphocytes. TSLP induces the differentiation of pro-allergic CD4 T-helper cells at the origin of the production of Th2 cytokines such as IL-4, IL-5 and IL-13 [72]. TSLP, acts as a key regulatory factor that induces type 2 inflammatory responses and as the other epithelium-derived cytokines IL-25 and IL-33, links innate and adaptive immunities [73]. 

TSLP mRNA and protein are elevated in the epithelial and infiltrating cells of patients with nasal polyps [28] and induce the production of Th2 cytokines in the nasal mucosa [29,30]. The production of TSLP by epithelial cells is controlled by both innate and adaptive immune signaling via activation of a protease receptor [74,75]. This suggests that TSLP plays a role in early activation at the initial site of exposure in the epithelium. Its direct role on epithelial cells and barrier function has yet to be described. TSLP blockers (Tezepelumab) could then represent an interesting option for future treatments [76]. 

## 3. Interleukin-1 Family Cytokines

### 3.1. Interleukin 1

IL-1α, considered as an alarmin and IL-1β, which is processed by the inflammasome, are immunoregulatory proteins secreted by activated monocytes, macrophages, natural killer cells, epithelial cells and fibroblasts, and they activate upstream the cascade of the inflammation process via a shared receptor [77]. Together with IL-6 and TNF, IL-1 belongs to the so-called “triade” of inflammatory cytokines. 

In CRSwNP, IL-1α is released from damaged epithelial cells, and further activates T-cells and monocytes. It upregulates the expression of adhesion molecules on monocytes such as intercellular adhesion molecule-1 (ICAM-1) and VCAM-1, which are essential to the recruitment of eosinophils [31,32]. IL-1β increased the secretion of CCL5 from nasal polyp fibroblasts, resulting in the attraction of monocytes, eosinophils and memory T-cells, and the recruitment and trans-endothelial migration of these cells to the inflammation site [33]. Besides its inflammatory effects, IL-1β is responsible for glucocorticoid resistance in nasal polyp tissue, rendering local treatment more complex [34].

Direct effects of IL-1 on epithelial cells and fibroblasts have yet to be described in CRSwNP.

### 3.2. Interleukin 33

IL-33 is a more recently described cytokine, secreted mainly by airway epithelial cells, endothelial cells, fibroblasts, macrophages and dendritic cells [78], and described as an “epithelial-derived alarmin” that activates the innate and humoral arms of the immune system in the presence of damage [79]. 

Several studies have reported upregulated expression of IL-33 and its receptor ST2 in nasal polyps at both the mRNA and the protein levels [80,81]. IL-33 expression in epithelial cells is induced via serine protease activity of *Aspergillus fumigatus* through PAR2 [82]. IL-33 mediates Th2-skewed eosinophilic inflammation, inducing the production of IL-4, IL-5 and IL-13 in an allergen-induced murine model [35]. Conversely, IL-33 possesses an antagonistic effect on Th17 inflammation [36]. IL-33 also plays a role in the limitation of neutrophil recruitment in CRSwNP. The quantity of neutrophils in an allergen-induced murine model of allergic rhinitis is significantly decreased after IL-33 treatment [83]. IL-33 is also involved in tissue remodeling: concentration of matrix metalloproteinase (MMP)-2 and -9 is positively correlated with IL-33 mRNA levels in nasal polyps, and it contributes to edema formation [37]. Moreover, IL-33 increases mucus overproduction in the eosinophilic inflammation of human airways [38].

## 4. Tumor Necrosis Factor-α

TNF-α is a pleiotropic cytokine possessing pro-inflammatory properties, produced by several cell types (epithelial cells, T-lymphocytes and macrophages), and it may induce the release of IL-6, IL-10 and INF-γ [84]. 

TNF-α mRNA and protein levels are increased in nasal polyps *versus* inferior turbinate tissues [41]. This cytokine is involved in the maintenance of inflammatory reaction through (1) the recruitment and accumulation of eosinophils via the upregulation in nasal polyps of the chemokine eotaxin and the adhesion molecule VCAM-1 [10,39,40] (2) the accumulation of CCL2, a monocyte chemoattractant, in fibroblast cultures derived from nasal polyps [41]. TNF-α serum levels are increased in all endotypes of CRSwNP [3]. 

## 5. Interleukin-6 Family Cytokines

### 5.1. Interleukin 6

IL-6 is produced by numerous types of cells, including T-cells, B-cells, monocytes, fibroblasts, epithelial and endothelial cells and tumor cells in response to micro-organisms or other cytokines (IL-1, TNF-β) [85]. IL-6 is a pleiotropic cytokine, well-known as a regulator of inflammation, classically described as inducing B-cell proliferation and activation and neutrophil recruitment [42]. It can exert its action via a dimeric receptor composed of the IL6-R and gp130 subunits, activating the STAT3 and MAP kinase pathways. IL-6 can also associate with its soluble receptor (sIL-6R), activating target cells which do not express membrane bound IL-6R but only gp130 by a process called “trans-signaling” [86]. Since all human cells express gp130 on their surface, the IL-6/sIL-6R complex can activate all cells in the body.

In CRSwNP, IL-6 mRNA and protein are overexpressed in nasal polyps compared to normal mucosa [87], probably by fibroblasts able to modulate the activation of immune responses (formation of plasma cells) and the synthesis of stroma. At the epithelial level, IL-6 increases sinonasal epithelial cell proliferation after epithelial damage and affects ciliary functions by increasing ciliary beating [43].

### 5.2. Oncostatin M

Oncostatin M (OSM) is an IL-6 family cytokine, characterized by the shared gp130 receptor subunit. OSM is produced by several cells of the immune system such as Th2 lymphocytes, eosinophils, neutrophils and macrophages. OSM is also a proinflammatory cytokine and, though less familiar than IL-6, OSM tissue effects are often more potent than the latter [88]. This cytokine targets epithelial cells, such as keratinocytes, by inhibiting cell differentiation and inducing chemokines and antimicrobial peptides [88]. OSM also plays an important role in pathologies associated with fibrosis such as systemic sclerosis, rheumatoid arthritis and interstitial pulmonary fibrosis, and has pro- or antifibrotic action depending on the model [89,90,91].

OSM protein and mRNA are overexpressed in nasal polyps compared to non-polypoid nasal mucosa in CRS. They can act on both the epithelial and fibroblastic components of the polyp tissue. On cultured epithelial cells derived from nasal sinus polyps, OSM causes an alteration of the epithelial barrier, evidenced by decreased transepithelial electrical resistance and increased permeability [44]. The effects of OSM on fibroblasts have recently been studied in CRSwNP. OSM counteracts the pro-fibrotic action of transforming growth factor-β (TGF-β1) on fibroblasts, by inhibiting myofibroblast differentiation and the production of extracellular matrix components.

## 6. Interleukin-17 Family Cytokines and Th17 Cytokines

### 6.1. Interleukin 17

IL-17A, a cytokine mainly produced by Th17 cells, is emerging as a critical player in host defense responses and inflammatory diseases. Skin, bronchial and intestinal epithelial cells are the main targets of IL-17. It activates signaling cascades leading to the induction of antimicrobial peptides and chemokines, further recruiting monocytes and neutrophils to the site of inflammation [45]. In vitro and in vivo data indicate that on keratinocytes, IL-17 acts synergistically with IL-1, IL-22, OSM and TNF-α to induce powerful inflammation [92,93]. IL-17 expression is linked to the pathogenesis of various autoimmune disorders, such as psoriasis [45].

IL-17 mRNA is overexpressed in nasal polyp tissue compared to normal nasal mucosa [94], and IL-17 is positively correlated to the eosinophil number in nasal polyp tissue [95]. In CRSwNP, Th2 and Th17 pathways regulate each other. Paradoxically, in Chinese patients whose infiltrate is mainly composed of neutrophils, Th2 cytokines IL-4 and IL-13 inhibit expression of IL-17 in homogenates of polyps tissue, whereas IL-17 enhances expression of Th2 cytokines [96]. The direct effects of IL-17 on epithelial cells and fibroblasts in this model is not yet known.

### 6.2. Interleukin 25

IL-25, also known as IL-17E, is a member of the IL-17 cytokine family, produced by sinonasal epithelial cells, mast cells and T-cells. IL-25 supports the Th2 immune response by stimulating IL-4, IL-5 and IL-13 production and decreasing IFN-γ production [97]. IL-25 also induces the recruitment of neutrophils via the stimulation of IL-8 production [98].

IL-25 mRNA expression is increased in nasal polyps compared to control tissue and promotes Th2 polarization of naive CD4 T-cells [46]. IL-25 can be secreted by nasal epithelial cells in response to allergens [99], helminth [100] and viral infection [101]. The release of this cytokine perpetuates eosinophilic inflammation in CRSwNP [47]. In mice, intraperitoneal or intranasal administration of IL-25 induces recruitment of eosinophils and Th2 cytokine production [102,103]. In the same model, anti-IL-25 treatment reduces polyp formation, mucosal thickness, collagen deposition and infiltration of inflammatory cells [46]. 

### 6.3. Interleukin 22

IL-22 is a member of the IL-10 family produced by various inflammatory cells such as Th17 and Th22, natural killer cells, eosinophils and mast cells [104]. IL-22 plays a role in regulating host defense, tissue homeostasis, cell differentiation and inflammation on epithelium and mucosa barrier surface [105,106].

In CRSwNP, IL-22 is positively correlated with type 2 immune mediators [48]. In a type 2 microenvironment, IL-22 initiates TSLP expression in airway epithelial cells [48]. Following exposure of dispersed nasal polyp cells to *Staphylococcus aureus* exotoxins, IL-22 positively regulates mucus production via the enhancement of MUC1 expression [49].

## 7. Type 1 Inflammatory Cytokines

Th1 cells promote cell-mediated immune response and are required for host defense against intracellular viral and bacterial pathogens. Th1 cells mainly secrete IFN-γ and IL-2. These cytokines promote macrophage activation, nitric oxide production, and cytotoxic T-cells proliferation, leading to the phagocytosis and destruction of microbial pathogens. Expression of Th1 cytokines is mainly found in patients with CRSsNP [107]. Although weakly expressed in nasal polyps, these cytokines play a role in the inflammatory cascade of CRSwNP.

### 7.1. Interferon-γ

IFN-γ is involved in a wide range of inflammatory processes and assumes diverse roles such as leukocyte attraction, activation of natural killer cells and regulation of B-cell functions. IFN-γ is secreted by Th1-lymphocytes, CD8+ cytotoxic lymphocytes, ILC1, natural killer cells and B-cells, and it promotes a Th1-biased environment [58]. 

Several studies have reported low levels of IFN-γ at protein levels in nasal polyps when compared to non-polypoid nasal mucosa [108,109]. Flow cytometry analysis of nasal polyps infiltrate has revealed a mixed Th1/Th2 profile with a significant population of IFN-γ^+^ Th1 cells, despite low protein levels detected in the homogenized tissue supernatants [110,111]. IFN-γ contributes to the recruitment of inflammatory cells such as eosinophils [59].

### 7.2. Interleukin 2

IL-2 plays a central role in T and B cells cooperation. It induces T-cell proliferation and secretion of IFN-γ and TNF-α. 

The levels of IL-2 measured by ELISA are decreased in nasal polyps compared to control tissue. There is a positive correlation between levels of IL-2 and proportion of Treg cells in nasal polyps, indicating that the decreased number of Treg cells in nasal polyps may result from the downregulation of IL-2 signaling pathway [60].

## 8. Regulatory T-Cell Cytokines

### 8.1. Interleukin 10

IL-10 is an anti-inflammatory cytokine that protects the host from excessive tissue damage during the host’s defense against pathogens [112]. IL-10 is mainly produced by Th2 cells, macrophages, B-cells, ILC2, dendritic cells, natural killer cells, mast cells and granulocytes [113]. Its main biological function is to inhibit the presentation of antigens by dendritic cells and macrophages and to inhibit the production of pro-inflammatory cytokines by Th1 lymphocytes such as IL-2 and TNF-α. Lastly, IL-10 decreases the synthesis and macrophagic secretion of metalloproteinases and inhibits the effect of IL-1 by releasing antagonists of its receptor [114,115]. In combination with TGF-β, IL-10 induces Ig switch toward Ig A production [116].

IL-10 mRNA and protein are upregulated in CRSwNP when compared with control tissue or CRSsNP [50]. Several pathogens develop mechanisms that up-regulate IL-10 during infection and create a more favorable pro-infectious environment. Staphylococcal protein A (SpA), a virulence factor from *S. aureus*, coupled to immunoglobulins in immune complexes, induces IL-10 production and suppresses staphylococcal enterotoxin B-induced IL-5, IL-13, IFN-γ and IL-17 production by nasal polyp cells [117]. Overexpressed in CRSwNP, IL-10 suppresses the production of pathogen-related inflammatory cytokines and contributes to reduced pathogen elimination favoring chronic inflammation [50]. 

### 8.2. Transforming Growth Factor-β

TGF-β is a crucial enhancer of immune homeostasis and tolerance, inhibiting the expansion and function of many components of the immune system. Besides its immunosuppressive properties, TGF-β has pro-fibrosing effects. TGF-β1 is produced by a variety of cells, including Treg lymphocytes, macrophages, eosinophils and fibroblasts. 

TGF-β1 is the main isoform detected in nasal polyps and has an anti-inflammatory action, reducing the synthesis of IgE and the activation of eosinophils [53,54]. The release of the chemokines MCP-1 and eotaxin by nasal polyp fibroblasts is also decreased after TGF-β1 stimulation [118]. Aside from its anti-inflammatory action, TGF-β allows the proliferation of fibroblasts and their differentiation into myofibroblasts. In addition to the expression of α-smooth muscle actin (α-SMA) allowing cell contractility, myofibroblasts produce many components of the extracellular matrix, such as fibronectin and type I and III collagens, which are important in tissue remodeling. On nasal epithelial cells, TGF-β induces abnormal differentiation [119,120] and promotes epithelial to mesenchymal transition via microRNA-21 (miR21) [56]. In CRSwNP, a strong expression of TGF-β1 and TGF-β2 proteins [121] is thought to be at the origin of epithelial remodeling and the process of fibrosis [55]. However, the role of TGF-β in the pathogenesis of nasal polyps has also been associated with downregulation of this cytokine. Studies have reported decreased levels of TGF-β in CRSwNP *versus* controls or CRSsNP [57]. Although spots of strong TGF-β labelling in focal fibrotic regions of nasal polyps can be identified, a relative absence of fibrosis and TGF-β staining is associated with the edema and pseudocyst formation characteristic of nasal polyps [57]. The downregulation of TGF-β may partially explain the relative upregulation of Th2 cytokines and persistent eosinophilia characteristics of nasal polyps. TGF-β1 serum levels does not differ between the CRSwNP clusters [3]. 

### 8.3. Interleukin 32

IL-32 is a recently described proinflammatory cytokine produced by T-cells, natural killer cells, monocytes, dendritic cells, endothelial and epithelial cells and fibroblasts [51,122], and has been reported to be associated with various inflammatory disorders such as CRSwNP [123,124]. 

IL-32 plays a role in responses to bacterial epithelial aggression. Indeed, LPS induces IL-32 mRNA and protein expression in nasal polyps-derived fibroblasts through the TLR4-JNK-AKT-CREB signaling pathway and the induction is greater in CRSwNP than in normal mucosa or CRSsNP [125]. IL-32 induces production of the proinflammatory cytokines TNF-α, IL-1, IL-6 and IL-8 by activating the signal pathways involving nuclear factor-κβ (NF-κβ) and p38 mitogen-activated protein kinase [51,52]. 

## 9. Conclusions

**Figure 1:**
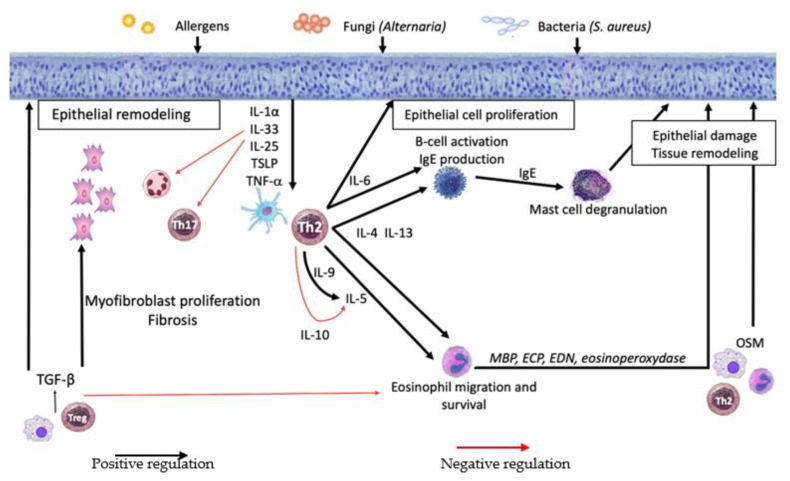
Summary of cytokines network involved in CRSwNP. OSM: oncostatin M; MBP: major basic protein; ECP: eosinophilic cationic protein; EDN: eosinophil-derived neurotoxin. Following an aggression of the nasal epithelium by *S. aureus*, *Alternaria* or allergens, a Th2 inflammatory response is induced by IL-1α, IL-33, IL-25, TSLP and TNFα. IL-6, IL-4, and IL-13 cause IgE production and mast cell degranulation, and IL-5 causes eosinophil activation and survival. These phenomena, associated with the action of OSM and TGF-β, lead to tissue remodeling characterized by alteration of the epithelium and fibrosis.

The pathophysiological concepts concerning the involvement of inflammatory cytokines in CRSwNP have gradually evolved. Although the central place of a Th2 cytokine environment, which is associated with an eosinophilic infiltration, retains a central role in the genesis of polyps, the role of other cytokine subpopulations has been recently highlighted. IL-1 family cytokines and TNF-α are involved early in the inflammatory cascade and, besides Th2 cytokines, allow recruitment of inflammatory cells (mainly eosinophils). IL-6 and OSM play an important role in tissue remodeling, especially in the alteration of the epithelial barrier and fibrosis. By inhibiting the production of pathogen-related inflammatory cytokines, IL-10 contributes to the maintenance of inflammation. Otherwise, TGF-β is central pro-fibrotic processes in CRSwNP. Taken together, these inflammatory cytokines reinforce a Th2 micro-environment leading to a specific and complex signature in CRSwNP (Figure 1).

The emphasis on cytokinic profile in CRSwNP has led to the use of biotherapies, including anti-IgE, anti-IL-5 and anti-IL-4Rα. While hindsight on biotherapies in other auto-immune diseases shows that non-responders or resistances are observed, it seems important to target other immune actors in CRSwNP as putative targets. Although additional cytokines have recently been characterized as overexpressed in CRSwNP, more research remains necessary to determine the molecular mechanisms involved and the specific downstream effects of these cytokines on inflammatory cells, epithelial cells and stromal tissue of nasal polyps, the long-term objective being to complete the arsenal of currently available biotherapies for the treatment of CRSwNP.

## Figures and Tables

**Table 1:** Production and action of cytokines over and under-expressed in chronic rhinosinusitis with nasal polyps.

Cytokines	Production	Action
***Cytokines over-expressed***		
IL-4	Th2 lymphocytes, eosinophils, basophils, natural killers and mast cells	Th2 polarization of naïve CD4 T-cells Activation of B-cells and production of Ig [7,8,9] Recruitment of eosinophils [10] Tissue fibrosis [11] Alteration of the epithelial barrier [12,13]
IL-13	Th2 lymphocytes, eosinophils, basophils, natural killers and mast cells	Recruitment of inflammatory cells Differentiation of monocytes into macrophages [14,15] Goblet cell hyperplasia [16] Recruitment of eosinophils [17] Alteration of the epithelial barrier [12]
IL-5	Th2 lymphocytes, masts cells and eosinophils	Recruitment and survival of eosinophils [18,19,20,21,22,23] Alteration of the epithelial barrier [24]
IL-9	Th2, Th9 lymphocytes and mast cells	Inducing IL-5Rα expression [25] Promoting the anti-apoptotic action of IL-5 in asthmatics [25] Production of proteases by mast cells [26] Release of IgE by B-cells [26] Favorizing a muciparous metaplasia [27]
TSLP	Epithelial cells	Th2 polarization of naive CD4 T-cells [28,29,30]
IL-1α	Epithelial cells, fibroblasts, monocytes, macrophages, natural killer cells	Recruitment of eosinophils [31,32]
IL-1β	Epithelial cells, fibroblasts, monocytes, macrophages, natural killer cells	Attraction of monocytes, eosinophils and memory T-cells [33] Glucocorticoid resistance [34]
IL-33	Epithelial cells, fibroblasts, macrophages, dendritic cells	Th2 polarization of naïve CD4 T-cells [35] Limitation of neutrophil recruitment [36] Edema formation [37] Mucus production [38]
TNF-α	Epithelial cells, T lymphocytes, macrophages	Recruitment of eosinophils [10,39,40] Recruitment of monocytes [41]
IL-6	T and B lymphocytes, monocytes, fibroblasts, epithelial and endothelial cells	Recruitment of neutrophils [42] Increased epithelial cell proliferation after damage [43]
OSM	Th2 lymphocytes, eosinophils, neutrophils, and macrophages	Alteration of the epithelial barrier [44] Antifibrotic action
IL-17	Th17 lymphocytes	Recruitment of monocytes and neutrophils [45]
IL-25	Epithelial cells, mast cells, T lymphocytes	Th2 polarization of naive CD4 T-cells [46] Production of eosinophils [47]
IL-22	Th17, Th22 lymphocytes, natural killer cells, eosinophils, epithelial cells	Initiation of TSLP expression [48] Mucus production [49]
IL-10	Th2 lymphocytes, B lymphocytes, macrophages, natural killers, ILC2, mast cells	Reduced pathogen elimination [50]
IL-32	T lymphocytes, natural killer, monocytes, dendritic cells, endothelial, epithelial cells and fibroblasts	Production of proinflammatory cytokines [51,52]
***Nonconsensual levels of expression***		
TGF-β	Treg lymphocytes, macrophages, eosinophils and fibroblasts	Reduced activation of eosinophils and proinflammatory cytokines [53,54] Proliferation of fibroblasts and myofibroblast differentiation [55] Epithelial to mesenchymal transition [56] Edema formation [57]
***Cytokines under-expressed***		
INF-γ	Th1 lymphocytes, ILC1, B lymphocytes, natural killer cells	Th1 polarization of naive CD4 T-cells [58] Recruitment of eosinophils [59]
IL-2	T lymphocytes	Treg polarization of naive CD4 T-cells [60]
